# Power decline and the change of self-esteem: The moderating effect of self-defense

**DOI:** 10.3389/fpsyg.2022.1052208

**Published:** 2022-12-21

**Authors:** Caiyun Huang

**Affiliations:** ^1^College of International Economics and Trade, Ningbo University of Finance and Economics, Ningbo, Zhejiang, China; ^2^Zhejiang Soft Science Research Base Digital Economy and Open Economy Integration Innovation Research Base, Ningbo, Zhejiang, China

**Keywords:** power decline, self-esteem, self-defense, self-enhancement, semi-structured interview

## Abstract

**Introduction:**

Power is a fundamental force in social relationships. Having more power means more freedom and resources and the ability to control and influence others. Psychologically, people are afraid of power decline, therefore are motivated towards self-enhancement to avoid the decline of self-esteem. We asked if power decline brings about a subsequent decline in self-esteem.

**Objective:**

To investigate whether power decline in social relationships leads to a decline in self-esteem and to explore the moderating role of self-defense.

**Methods:**

A laboratory experiment was conducted with college students in East China as subjects, which was divided into manipulation tests of power decline and Self-Defense (*N* = 61) and two formal experiments (*N* = 65; *N* = 160). In addition, a semi-structured in-depth interview was used to further improve the ecological validity of the findings.

**Results:**

(1) Power decline did not lead to a decline of self-esteem, and self-esteem rises when power remained unchanged; (2) When the level of self-defense was higher, constant power lead to a greater increase of self-esteem, and the decline of power would not lead to the change of self-esteem; (3) When the level of self-defense was lower, the relationship between constant power and the rise of self-esteem was weakened, and power decline would not lead to the significant change of self-esteem. At the end of this study, the theoretical and practical implications are discussed.

## Introduction

Power is a fundamental force in social relations (Russell, 1938) and plays a crucial role in many areas, such as economics, politics, and general social interaction processes, penetrating almost every corner of organizations. Power is often understood as the control over money, information, or decisions (Galinsky et al., 2003) or influence over the thoughts and behaviors of others (Keltner et al., 2003). Individuals’ perception of their own power has a greater impact on behavior than the power they actually have (Haidt and Rodin, 1999). In our study, we define power to means self-perceived power. Psychologists generally agree that feeling powerful (self-perceived power) leads in turn to higher feelings of self-esteem (Anderson et al., 2012; Kuehn and Gordon, 2015; Wang, 2015), which is defined as a positive or negative attitude toward the self (Rosenberg, 1965) and is one of the central constructs of personality (Bolognini et al., 1996). The reason for the positive relationship between self-perceived power and self-esteem is because possessing power means access to higher levels of resources and freedom, also lower levels of personal risk (Galinsky et al., 2015), which in turn leads to a more positive self-evaluation, i.e., the individual’s self-esteem. To better understand the subsequent effects of power, it is necessary to clarify its impact on the ego.

The refinement of rules and institutions has made people more attracted to power and risk averse towards any threat that would result in a loss of power (Anderson and Brion, 2014). However, declines in power often occur, such as dismissal, demotion, kick-upstairs (defined as someone being apparently ascended but actually descended), and retirement. At this point, the individual loses the ability to control and influence others. This loss of power means the loss of resources, freedom to act, and can also result in a loss of goal orientation (Guinote, 2017). They lose the objective conditions and psychological capital to continue to self-actualize without consequences, to overestimate themselves without repercussions, and to feel important and superior to others (Keltner et al., 2003). In addition, loss of power predicts the loss of legitimizing privileges (Wojciszke and Struzynskakujalowicz, 2007) and potentially the respect of other powerful peers. All of these indicate that loss of power can lead to the loss of conditions that maintain self-esteem. However, a study by Sivanathan et al. (2008) found that after power decline, individuals often behaved as if they had not lost power. So, does power decline really bring about a decline in self-esteem?

According to the theory of self-enhancement, although the objective conditions for maintaining high self-esteem are lost due to the decline of power, self-enhancement motivates the individual to strive to maintain a positive self-perception (Kobayashi and Brown, 2003). This intertwined state of self-esteem, self-protection, and desire for self-improvement results in a virtual personal zoo of self-defense mechanisms (Tesser, 2001). The purpose of such self-defense mechanisms is to maintain and increase self-esteem (Crowell et al., 2015). Therefore, when power declines, individuals do not allow their self-esteem decline, but rather attenuate the negative effects of declining power through various means of personal self-improvement. This study further explores the role of self-defense in the effects of declining power on self-esteem. The higher the level of an individual’s self-defense, the more sensitive he or she is to factors affecting self-concept, which in turn moderates the effect of declining power on self-esteem (Waqas et al., 2015).

The theoretical contributions of this study are as follows: firstly, most previous research on power has been conducted from the perspective of static power and power threat, instead of power change, especially a decline in power. This study makes up for the lack of research in this area, by deepening the understanding of the effect power has on the psyche, and thereby expanding the scope of power theory. Secondly, by drawing on the methods of power manipulation, the contextual simulation of the human response to power change paves the way for future research on power change; Thirdly, by studying the question if a perceived decline in power also brings about a decline in self-esteem, this study provides a new perspective on the relationship between power and self-esteem, which broadens power theory. Finally, the boundary effect of self-defense is explored to further validate the role of self-enhancement motivation in power decline events.

## Theory and hypotheses

### Power decline and self-esteem decline

Compared to the powerless, powerful people have more freedom, less risk, higher positive emotions, more freedom to engage in arbitrary thoughts, and higher self-esteem (Kipnis, 1972; Keltner et al., 2003). Power decreases results in the inability to influence others at will and increased limitations (Keltner et al., 2003). The individual also is aware of their inability to continue to have an impact on others and the outcome of events. A decline in power means they no longer have the objective conditions and psychological capital to continue to freely utilize their abilities, to feel superior to others or to criticize others unchallenged. That is, positive self-perceptions and evaluations are diminished, which leads to a decline in self-esteem. In addition, when an individual loses power, he or she also loses legitimate privilege and others may no longer consider the physical and social capital he or she currently possesses to be deserved (French and Raven, 1959). The individual’s inability to continue to be respected by others can reduce the individual’s self-confidence, which can result in a decrease in self-esteem. Overall, the loss of power impairs the individual’s self-worth, affects his or her positive self-evaluation and judgment, and deprives him or her of the conditions for maintaining self-esteem.

According to the theory of self-enhancement, to maintain their ego individuals need to increase their self-worth and self-esteem, as well as a need to seek positive self-perceptions and avoid negative evaluations, which manifests as a tendency to maintain an unrealistically positive self-concept and encompasses self-enhancement and self-protection, with the latter being predominant (Sedikides and Gregg, 2006). The tendency to self-protection will be stronger in high-powered individuals (Kobayashi and Brown, 2003). When their power decreases, although they will lose the objective conditions and psychological capital to maintain their self-esteem, they will have a strong need to protect their threatened self-esteem as a way to maintain their positive self-image. As a result, their self-esteem level will not change significantly. Therefore, self-esteem strives to maintain at its original level even after the decline of power. When power is constantly high, there is a temporal comparison between the individual’s before and after self-evaluation, and self-improvement is often achieved by devaluing the past self and exaggerating the present self (Festinger, 1954). Self-improvement will bring individuals higher positive self-images and self-esteem. Therefore, this study hypothesized that:

*H1a*: Constantly high power is positively related to self-esteem increase.

*H1b*: Power decline is not related to self-esteem decline.

### Self-defense as a boundary condition

Ego-defense mechanisms are how individuals subconsciously distort threatening information to preserve certain thoughts or feelings (Waqas et al., 2015), including two approaches: defense (negative) and coping (positive). The purpose of such mechanisms is mainly to maintain and increase self-esteem (Crowell et al., 2015). The manifestation of individual defense mechanisms is also a form of personality (Huang et al., 2018). Individuals with high self-defense are more sensitive to threatening events to the self-esteem (Popelnukha et al., 2022). With power as an important factor affecting self-esteem, individuals with high self-defense would become more responsive to power altering events. At the same time, their need to enhance self-esteem, increase self-worth, and psychologically concentrate on positive self-perceptions, while suppressing negative evaluations is more urgent (Popelnukha et al., 2022). Thus, when power decreases, individuals with high self-defense tendencies will lose the objective conditions for maintaining self-esteem, but they will try more intensively to protect their ego from damage and maintain their self-esteem at the previous level. When power remains constantly high, individuals with high self-defense will increase their internal temporal comparison of self-appraisal (Wilson and Ross, 2001) and produce a higher level of self-improvement, i.e., bring about higher self-esteem. Combined with Hypothesis 1a and Hypothesis 1b, the present study hypothesized that when individuals have a higher tendency towards self-defense, their change in self-esteem will be stronger with changes in power. Therefore, the study hypothesized that:

*H2a*: Self-defense positively moderates the relationship between constantly high power and self-esteem increase. The positive relationship between constantly high power and self-esteem increase would be strengthened when self-defense is high, and vice versa.

*H2b*: Self-defense does not moderate the relationship between power decline and self-esteem decline. The relationship between power decline and self-esteem decline is not significant whether self-defense is high or not.

Based on self-enhancement theory and power theory, this study explored whether a decline in power brings about a decline in self-esteem. A manipulation test of power decline and self-defense was conducted through a pre-experiment to validate the main effect in study 1 and the moderating effect in study 2. In addition, we used a semi-structured interview to improve the ecological validity of the findings in study 3.

## Pre-experiment

### Manipulation test of power decline

Drawing on Sivanathan et al.’s (2008) research, this study used a contextual simulation that integrated a dictator experiment and an ultimatum experiment to assess individuals’ sense of power as a way to determine whether differences in power across contexts were significant.

#### Procedure

Sixty-one undergraduate students from East China volunteered to participate in the experiment, 22 male and 39 female (*M* = 1.64, *SD* = 0.48).

Subjects were asked to read the contents of different situations and make an allocation plan and judge the sense of power as required. A total of eight scenarios were set up based on the amount of money, whether the subordinate was informed, and whether the subordinate could refuse. The specific scenarios were described as follows: “Imagine that you are a project manager of a company and you need to work with a subordinate to complete an engineering project, and you are required to allocate the project funds ($100,000/1 million in total). The subordinate knows (or does not know) the total amount of this funding, and the subordinate has the right to reject the allocation proposal. Once rejected, the project will be called off and you, as the project manager, will be demoted (or the subordinate will have to accept whatever the allocation plan is).

To prevent subjects from guessing the purpose of the experiment and the possible interference of the preceding and following situations, two questions of the Raven’s Intelligence Test were inserted between each situation. A total of 15 questions were selected.

The measure of personal power was selected from the scale developed by Anderson and Galinsky (2006), and two questions with a 5-point scale were selected. In this experiment, all the internal consistency coefficients of the eight scenarios’ measures were greater than 0.70.

#### Results

The results of the repeated measures ANOVA showed that the main effect of power manipulation was significant, *F* (1, 60) = 1323.39, *p* < 0.001, η_p_^2^ = 0.96. The results of the t-test showed that subjects’ sense of power was significantly higher in the case of subordinates who could not be refused than in the case of subjects who could be refused when the other two conditions were consistent. 100,000 + subordinates informed: could not be rejected (*M* = 3.19, *SD* = 0.88) was significantly higher than could be rejected (*M* = 2.66, *SD* = 0.77), *t* = 4.43, *p* < 0.001, Cohen’s *d* = 0.56; 1 million + subordinates informed: could not be refused (M = 3.28, SD = 0.84) was significantly higher than could be refused (*M* = 2.71, *SD* = 0.83), *t* = 4.41, *p* < 0.001, Cohen’s *d* = 0.57; 100,000 + subordinates not informed: could not be refused (*M* = 3.30, *SD* = 0.89) significantly higher than could be refused (*M* = 2.80, *SD* = 0.81), *t* = 4.31, *p* < 0.001, Cohen ‘s *d* = 0.55; 1 million + subordinates not informed: could not be refused (*M* = 3.46, *SD* = 0.93) significantly higher than could be refused (*M* = 2.85, *SD* = 0.79), *t* = 4.76, *p* < 0.001, Cohen’s *d* = 0.61.

The sense of power in the $1 million case was higher than the sense of power in the $100,000 case only when the subordinate was unaware and could not refuse, *t* = 2.71, *p* < 0.01, Cohen’s *d* = 0.34. In all other cases (subordinate informed + could be refused, subordinate informed + could not be refused, subordinate not informed + could be refused), the assigned amounts did not cause significant differences.

Only when the subordinate can refuse +1 million, whether the subordinate is informed or not does not cause a significant difference in the sense of power. In all other cases, the difference was significant, 100,000 + subordinate could not refuse: subordinate not be informed was significantly higher than subordinate be informed, *t* = 2.27, *p* < 0.05, Cohen’s *d* = 0.30; 1 million + could not be refused: subordinate be not informed was significantly higher than subordinate be informed, *t* = 2.65, *p* < 0.05, Cohen’s *d* = 0.34; 100,000 + could be refused: subordinate be not informed significantly higher than subordinate be informed, *t* = 2.07, *p* < 0.05, Cohen’s *d* = 0.23.

In summary, all three conditions can cause individual power differences to some extent. To obtain the maximum effect of power decline, all three conditions were included in the contextual setting.

### Manipulation test of self-defense

The manipulation of Self-defense drew on Huang et al.’s (2018) approach, using a self-affirmation task for initiation. Participators should complete in 8 min.

#### Procedure

Forty-six undergraduate students from East China volunteered to participate, including 22 males and 24 females (*M* = 1.52, *SD* = 0.51).

Subjects in the self-affirmation group (*n* = 24) were required to recall and write down two of their specialties and the corresponding scenarios as requested. Subjects in the control group (*n* = 22) were required to write down two significant inventions and their contributions as requested. Subsequently, the subjects rated their level of self-defense.

For the measurement of self-defense, the DSQ, a self-defense style questionnaire developed by Bond et al. (1983), was selected with six items and a 9-point scale. The internal consistency coefficient was 0.70.

#### Results

The results of the independent samples t-test found that the self-affirmation group had a significantly higher self-defense score (*M* = 5.46, *SD* = 1.17) than the control group (*M* = 4.80, *SD* = 0.88), *t*(44) = 2.16, *p* < 0.05, Hedges’ *g* = 0.63. Thus, the contextual initiation of self-defense was effective.

## Study 1 the decline in power and the decline in self-esteem

### Procedure

We recruited 65 undergraduate students, 22 males and 43 females (*M* = 1.66, *SD* = 0.48), in East China. They were all voluntary and rewarded with a cash prize.

Two groups were divided: the power decline group (32) and the power constant group (33). Applying the three conditions simultaneously, the reading material presenting the initial power (the highest power) to the subjects was as follows: “... Project funding (totaling $1 million) was allocated. The subordinate has no way of knowing the total amount of this funding, but the subordinate will have to accept whatever the allocation plan is.”

After the allocation of the $1 million, the subject rated his or her self-esteem.

Subsequently, subjects were required to view a short video (5 min or less) and answer a question, and only those who answered correctly could continue to start the following experiment. This was done in order, first, to judge whether the subjects were serious about the experiment; second, to prevent the subsequent experiment from being interfered with by the previous answers; and third, to relieve the subjects’ fatigue and nervousness. Through this process, 7 subjects were removed, leaving 58 subjects at the end, with a total of 28 subjects in the power decline group (8 males and 20 females, *M* = 1.71, *SD* = 0.46) and 30 subjects in the power constant group (9 males and 21 females, *M* = 1.70, *SD* = 0.47).

After completing the video task, the subjects again read a written piece about power, and the decreasing power group read the following material: “... the project just started running not long ago, and the company has allocated another sum of money to you (100,000 in total), which again needs to be distributed by you, but the subordinates know the amount of money and has the right to refuse. If the subordinates refuse, the project will be stopped and you, as the project manager, will be demoted.” The material read by the constant power group was as follows: “... the project has just started running not long ago, and the company has allocated another sum of money to you (1 million in total), which again needs to be distributed by you. The subordinate also has no way of knowing the total amount, and no choice but to accept whatever the distribution plan is.” The participators made the allocation, their self-esteem levels were evaluated, thereafter the experiment ended.

Self-esteem was measured using the Self-Esteem Scale developed by Rosenberg (1965), with 10 question items and a 4-point scale. The internal consistency coefficients of this scale in this study were 0.82 for initial self-esteem and 0.85 for second self-esteem. The internal consistency coefficients of pre-and post-self-esteem in the declining power group were 0.78 and 0.86, respectively; the internal consistency coefficients of pre-and post-self-esteem in the constant power group were 0.80 and 0.83, respectively.

### Results

First, the scores of self-esteem before and after in the two groups were compared. The results showed that no significant decrease in self-esteem occurred for subjects in the power decline group, *t* (27) = 0.80, *p* > 0.05. A significant increase in self-esteem occurred for subjects in the power constant group, before: *M* = 2.78, *SD* = 0.38; after: *M* = 2.84, *SD* = 0.38, *t* (29) = −2.26, *p* < 0.05, Cohen ‘s *d* = 0.17. Thus, it is clear that a sustained high power state boosts individuals’ self-esteem.

**Figure 1:**
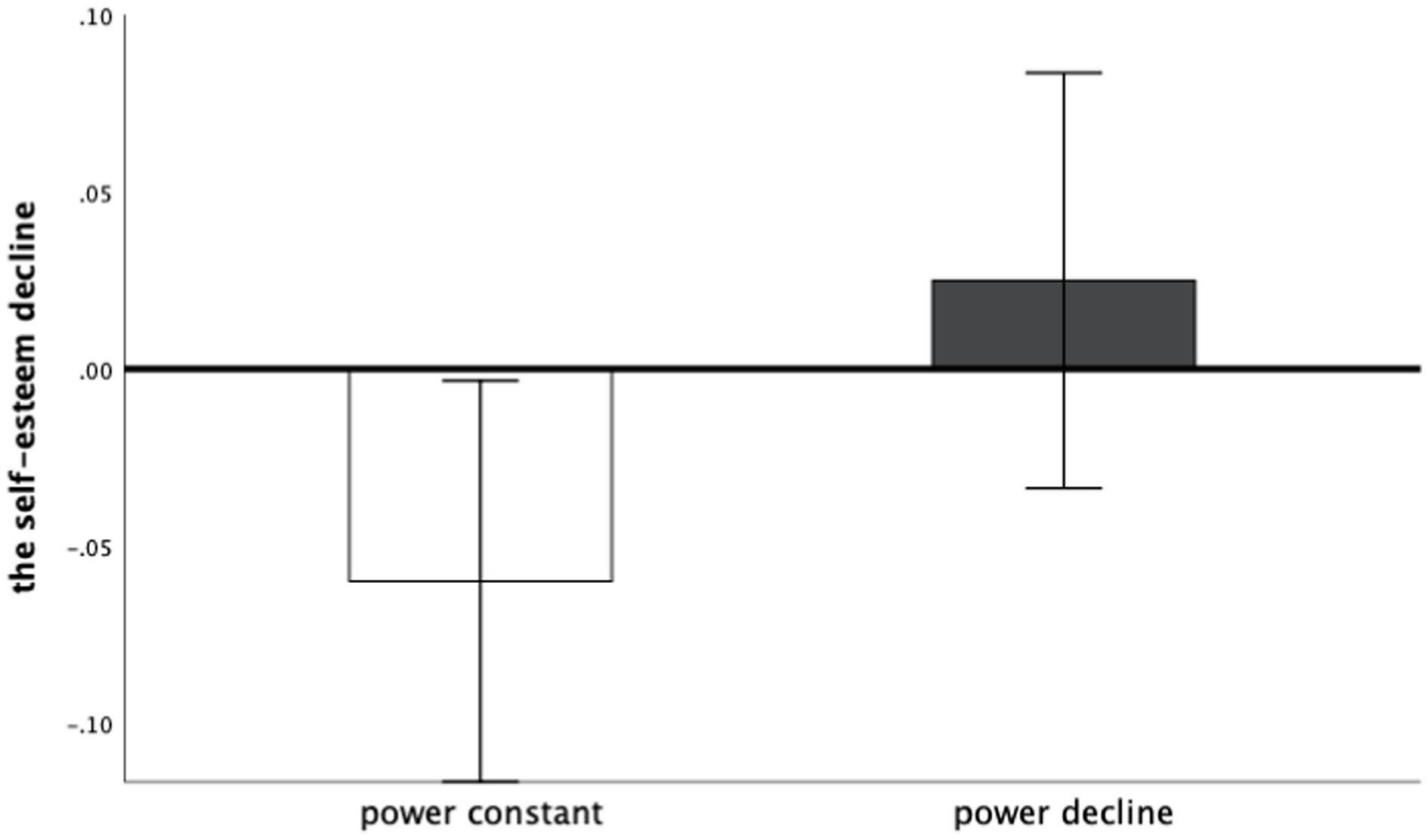
The self-esteem decline of different groups of Study 1.

The levels of change in self-esteem were compared between the two groups of subjects. There was a significant difference in the level of self-esteem decline between the power decline group and the power constant group, *t* (56) = −2.09, *p* < 0.05, Hedges’ *g* = 0.55. It can be seen that the self-esteem in the power constant group was significantly increased, while the self-esteem in the power decline group did not change significantly. It can be seen that although a decrease in power does not cause a decrease in self-esteem, it mitigates the increase in self-esteem brought about by constant power (persistently high power), and hypothesis 1a and hypothesis 1b were confirmed. See Figure 1.

### Discussion

A direct comparison of pre-and post-self-esteem levels in the power decline group did not show significant results; however, the pre-and post-self-esteem levels in the power constant group were significantly higher, and the results verified the hypothesis 1a and hypothesis 1b. The increase in self-esteem in the power constant group gives us a deeper understanding of power. The constantly high power does not increase the individual’s perception of power, but it will emphasize the state of high power, which in turn brings about a rise in self-esteem. This shows that individuals are sensitive to power signals (Sivanathan et al., 2008). The mere presence of a power signal, even if it is only the same level of power as before, will prompt individuals to recognize and reinforce the power they already possess. This temporal comparison effect could be a direction for future longitudinal studies of power. Although numerous studies have confirmed that power positively predicts individuals’ self-esteem (Wang, 2015; Wang et al., 2018), subjects in the power decline group did not change their self-esteem levels, suggesting that the power decline is not simply a shift from high to low power and that individuals’ self-esteem is not affected.

## Study 2 the moderation of self-defense

### Procedure

160 undergraduate students, 50 males and 110 females (M = 1.69, SD = 0.46) were recruited from Eastern China. They were all voluntary and rewarded with a cash prize.

They were divided equally into four groups of 40 students each: constant power + non-self-defense group, constant power + self-defense group, declining power + non-self-defense group, and declining power + self-defense group.

The only difference from the Experiment 1 process: the initiation of self-defense was performed before the second self-esteem measure.

A total of 21 people were removed through the mini-video. The final numbers left in each group were: 11 males and 24 females in the constant power + non-self-defense group (*M* = 1.69, *SD* = 0.47); 13 males and 22 females in the constant power + self-defense group (*M* = 1.63, *SD* = 0.49); 5 males and 30 females in the declining power + non-self-defense group (*M* = 1.86, *SD* = 0.36); and 10 males and 24 females in the declining power + self-defense group was (*M* = 1.71, *SD* = 0.46).

The internal consistency coefficients of the self-esteem scale in the study 2 were 0.79 (initial self-esteem) and 0.77 (second self-esteem). The internal consistency coefficients of the pre-and post-self-esteem in the constant power + non-self-defense group were 0.84 and 0.80, respectively. The internal consistency coefficients of the pre-and post-self-esteem in the constant power + self-defense group were 0.73 and 0.70, respectively. The internal consistency coefficients for the pre and post self-esteem in the declining power + non-self-defense group were 0.79 and 0.79, respectively. The internal consistency coefficients for the pre and post self-esteem in the declining power + self-defense group were 0.75 and 0.77, respectively.

### Results

None of the homogeneity of variance test results was significant, so these results are suitable for ANOVA. The main effect of power change on self-esteem change was significant, *F* (1, 137) = 7.03, *p* < 0.01, η_p_^2^ = 0.05. The subjects’ second self-esteem level was significantly higher (*M* = 2.87, *SD* = 0.40) than the first self-esteem level (*M* = 2.80, *SD* = 0.43) when power was constant, *t* (69) = 3.80, *p* < 0.001, and Cohen’s *d* = −0.62. The difference between the subjects’ two self-esteem levels was not significant when power declined, *t* (68) = 0.00，*p* > 0.05. The main effect of self-defense on the decrease in self-esteem was not significant, *F* (1, 137) = 0.09, *p* > 0.05. This result again verified the conclusion of experiment 1 that a decrease in power does not bring about a significant decrease in self-esteem, but a significant increase in self-esteem with constant power. Hypothesis 1a and hypothesis 1b were again verified.

**Figure 2:**
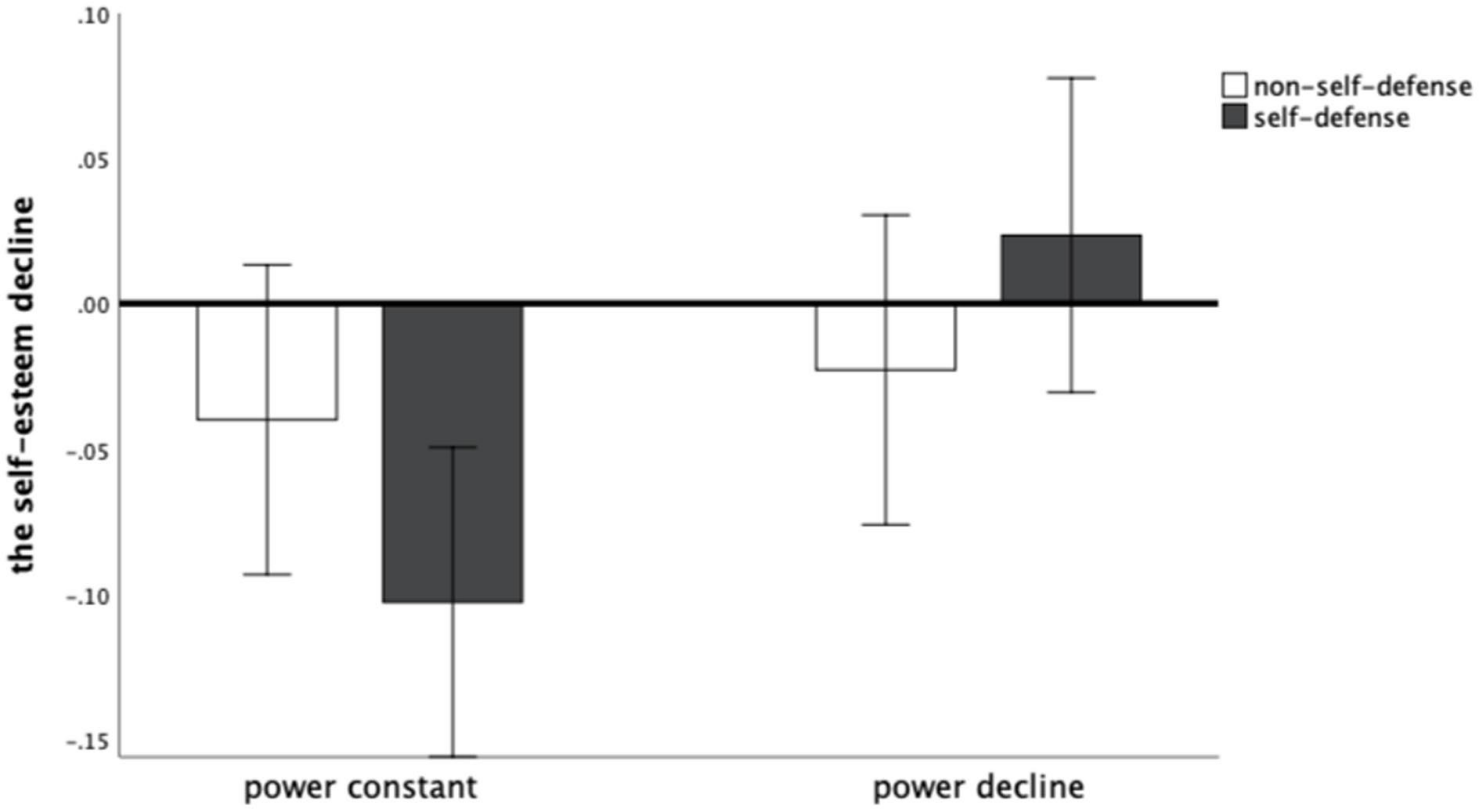
The self-esteem decline of different groups of Study 2.

The interaction between declining power and self-defense on the decrease in self-esteem was significant, *F* (1, 137) = 4.07, *p* < 0.05, η_p_^2^ = 0.03. When the self-defense was low, the difference in the self-esteem decrease between constant power (*M* = -0.04, *SD* = 0.12) and decreasing power (*M* = -0.02, *SD* = 0.13) was not significant, *t* (68) = − 0.58. When the self-defense is high, the difference between the self-esteem decrease in the case of constant power (*M* = -0.10, *SD* = 0.19) and the case of decreasing power (*M* = 0.02, *SD* = 0.19) is significant, *t*(67) = −2.77, *p* < 0.01, Hedges’ *g* = 0.67. Hypothesis 2a and hypothesis 2b was tested. The specific effects can be seen in Figure 2.

### Discussion

The results of the current study showed that a decrease in power does not bring about a decrease in self-esteem, but a constant power brings about an increase in self-esteem. In addition, the interaction effect between power decline and self-defense on decline in self-esteem is significant. When individuals have higher self-defense, the rise in self-esteem in the constant power state will be more pronounced, and the decline in self-esteem in the declining power state will not be significant. The results are consistent with the hypotheses. Higher levels of defensiveness mean more sensitivity to events that affect self-esteem (Popelnukha et al., 2022) and contribute to the impact of events on self-esteem. Conversely, individuals with lower levels of defensiveness are insensitive to events that result in power decline, which leads to a weaker impact on self-esteem. Thus, for those individuals with higher self-defense, the power decline elicits a stronger response, resulting in a greater impact on self-esteem.

## Study 3 semi-structured in-depth interviews

To further validate and enrich the research model and improve the ecological validity of the findings, study 3 used the typical sampling principle to select Employee A of a technology R&D department in a machinery industry as a case study subject, and used unstructured in-depth interview to conduct in-depth interviews about the background, passage, causes, and results of Employee A’s power decline, as well as Employee A’s feelings and experiences throughout the process. This case study approach can contribute to a greater overall understanding of the entire process of power decline and the environmental characteristics and outcomes of its effects (Merriam, 1998), and to some extent compensate for the lack of authenticity of laboratory experiments, as well as validate the model of the study, and provide fuller support for the reliability of the conclusions.

### Selection of the interviewee

In the process of selecting interviewees, we found a difficult reality that most of the people chose to leave their original work environment before or after the decline in power events, and the time point of the event is too far in the past to obtain accurate information. After screening, employee A was selected as an example for the following reasons: the time point of the decline in power was relatively recent (started 6 months ago and lost power completely after 3 months), and he still works in the company’s department after the decline in power occurred. He has worked in the company for 10 years and has a strong emotional commitment to the organization, and reacts strongly to the decline in power.

### Interview outline

Based on the research model, the outline of the questions revolved around the five set areas, allowing Employee A to describe them in as much detail as possible. Different from structured interviews, semi-structured in-depth interviews are designed to obtain the maximum amount of information by fully communicating with the individual and making appropriate adjustments to the outline during the interview process. Therefore, the questions in the interview outline are not standardized topics.

The five areas of the interview outline are.

1. the whole process of the power decline event.
2. the individual’s feelings of self-esteem after the event.
3. the individual’s feelings of self-esteem before the event.
4. whether self-defense was carried out after the event.
5. the effect on self-esteem after self-defense occurs.

During the interview, with the consent of Employee A, nearly 40 min of recorded interview material was obtained. After transcribing it into text, a total of 5,000 words of textual material was obtained.

## Results and discussion

Employee A’s reaction to his loss of power is generally stronger. Although he recognized the newcomer who replaced him, he was very disappointed and angry at the company. After all, he had worked in the company for 10 years and was considered a veteran employee. And the reason for the company to decrease his power is that the employee was having difficulty fully taking care of their work and life responsibilities over a short period of time, not because of a lack in ability or competence. This is cold-blooded. He has always recognized his competence and believes that his self-esteem has declined, but not significantly. This study combined Employee A’s own words, and the definition of self-esteem, to conclude that the matter of declining authority did not significantly reduce Employee A’s level of self-esteem.

Employee A’s statement that he is a person with an extremely high level of self-esteem is confirmed by his recognition of himself as a person who joined the technical department of the company after graduation and whose working ability has been continuously recognized, thus strengthening his self-recognition and gaining the corresponding power. The study concluded that Employee A’s 10 years of work experience had strengthened his self-esteem, and his “spiritual leadership” position after gaining power had strengthened his belief that he was a capable and successful person. Therefore, in this case a sustained high-power state does lead to an increase in self-esteem levels.

Employee A complains to his ex-colleague after his power decreases and has an intentional conflict with his manager. In addition, he also analyzes and explains the reasons for the decrease in power and produces more regressive behaviors (e.g., indulging in recreational activities, shopping, etc.), all of which indicate that he adopts a higher level of self-defense. According to our findings, individuals with high self-defense have their self-esteem affected by a decrease in power, which is reflected in the fact that self-esteem is no longer elevated or even has a slight decrease. Employee A experienced a non-significant decrease in self-esteem level, which is consistent with our results.

## General discussion

### The discussion of results

By drawing on and modifying Sivanathan et al.’s (2008) power change manipulation, our study used a scenario simulation with three power-related variables to allow participators to self-assess their sense of power in the situation. In this way, the subjects’ different sense of power in different situations were judged, and the results of the manipulation test confirmed that this scenario-simulated dictator and ultimatum game were effective and could indeed cause a decrease in individual power. Study 1 tested hypothesis 1a and hypothesis 1b: there was no significant change in the pre-and post-self-esteem in the power decline group; the pre-and post-self-esteem in the power constant group, however, increased significantly. This shows that even if power remains constant, the continuous presence of this power signal will promote individual self-esteem. Previous studies have suggested that power levels positively predicted self-esteem levels (Wojciszke and Struzynskakujalowicz, 2007; Anderson et al., 2012; Kuehn and Gordon, 2015) and that high power holders experience more objective conditions to maintain self-esteem. At the same time, the high stability of self-esteem (Kuster and Orth, 2013) and the strong resistance to the decline of self-esteem results in individuals maintaining their positive self-images (Kobayashi and Brown, 2003). Thus, the impact of power decline on individuals is not simply a linear path from “high power” to “low power,” but a more complex process, and it is important to explore the contents of this black box.

Study 2 tested Hypothesis 2a and Hypothesis 2b: Self-defense moderates the relationship between power decline and self-esteem decline. When power is constant, a more significant increase in the self-esteem level of high self-defense individuals occurs; when power decreases, the self-esteem level of high self-defense individuals does not change significantly. Self-defense, as a stable personality trait, represents the individual’s sensitivity to information that threatens the self and self-esteem (Popelnukha et al., 2022). In this study, for individuals with high self-defense, whether power decrease or not would have bigger different impacts on self-esteem. Most of the previous studies have explored self-defense as a mechanism and less as a personality trait (Huang et al., 2018), so there is no way to understand why various individuals have different intensities of defense in the face of threatening events. The present study begins with self-defense tendencies to understand their moderating effect on the relationship between power decline and self-esteem decline. This exploration of boundary conditions not only deepens the understanding of power decline, but also provides support for the idiosyncratic nature of self-defense.

### Theoretical implications

Our findings have at least four theoretical implications. First, most current research on power has focused on static power, and not much research has explored changes in power levels, especially power decline (Sivanathan et al., 2008). Research exists on the positive relationship between power and self-esteem (e.g., Fiske, 1993; Keltner et al., 2003; Anderson et al., 2012; Kuehn and Gordon, 2015; Wang, 2015), that is more power would lead to higher self-esteem. Jordan et al. (2011), Mooijman et al. (2019), and Wisse et al. (2019) explored the impact of power stability. Yong et al. (2010), Pettit et al. (2016), and Reh et al. (2018) found individuals would initiate active measures to compensate for power loss in the face of status threats. That research however can only show that individuals are averse to power loss. Little research has examined what effects constantly high power and power decline have on the change of self-esteem. However, the widespread existence of power decline in real-life situations and its effects on individuals’ minds and bodies have prompted researchers to explore this area. Our study can, to some extent, fill this gap and deepen the understanding of the concept and the theory of power and expand the outreach of power research.

Second, by drawing on the methods of power manipulation, the contextual simulation of power change can better reflect the real sense of power. The study used a contextual simulation of self-assessment to better reflect the real sense of power. At the same time, we introduced three contextual variables (the amount of distribution, whether the recipient was informed, and whether the recipient could refuse) simultaneously in the formal experiment to maximize the effect of power change. This kind of power change research using contextual simulation can provide an methodological concept and a new direction for future research, and opens the door for experimental research on power change.

Third, focusing on the core of the entire personality, the study tried to answer the question “Does a decline in power bring about a decline in self-esteem?” Motivation to self-promote is one of the main motivations in self-motivation (Leary, 2007). So based on the power theory and the theory of self-enhancement, we provide a new perspective on the relationship between power decline and self-esteem change, which can provide the most direct explanation of the effect of power decline and provide a basic direction for subsequent research.

Fourthly, the exploration of the boundary effects of self-defense fully explains why the effects of power decline are greater for some people, while some people respond more moderately to power change. Self-defense is an important ego-defense mechanism which helps protect certain thoughts or feelings (Waqas et al., 2015), especially self-esteem (Crowell et al., 2015). This exploration of individual differences can provide more explanations for the impact of power decline, consolidate the strength of the theory’s explanation of the model, and deepen the understanding of the theory.

Finally, the study not only deepens the understanding of individual power but also expands the results of socio-economic power, such as privilege and discrimination. Research confirms that social power is a cornerstone of racism, sexism, and privilege (e.g., Operario and Fiske, 1998; Fisher and Hammond, 2018; Rankin et al., 2021). The results of this study show that loss of power does not result in a decline in self-esteem because of the personal need for self-enhancement which the individual maintains through an active psychological process. However, it does not mean power decline has no effects on the privileged in that self-esteem becomes stable instead of inflated (under constantly high power). This further confirms that powerful individuals tend to actively work against power decline (Van de bos, 2009; Yong et al., 2010; Reh et al., 2018; Mooijman et al., 2019; Wisse et al., 2019), and even pretend that their power position has not changed (Sivanathan et al., 2008). This study examined the short-term effects of power decline using experimental methods, which could not verify long-term effects. Therefore, it remains to be further tested whether the findings can be extrapolated to long term conditions. Compared to the powerful, the self-esteem of the powerless is low (Anderson et al., 2012; Kuehn and Gordon, 2015; Wang, 2015). After a period of power decline, the privileged stabilize at a lower power level and their self-esteem at this state would be expected to have decreased. On the other hand, they may strive to regain power as a coping mechanism through some means, such as increasing oppression, and abuse, or harming the powerless (Rankin et al., 2021). So the further illumination of the effect of power decline on the privileged provided by these results points out new directions for the study of power, prejudice, discrimination, and privilege.

### Practical implications

The findings of this study have the following implications for real world scenarios: First, the aftermath of demotions can hinder the continual rise of individual self-esteem, which would require organizations or leaders to work in opposition to these tendencies by proactively enhancing employees’ self-esteem by affirming their abilities and values more often at the same time or shortly after a demotion. If the organization informs the whole company of the decision or the leader reprimands the employee when informing the news, it will further negatively affect the employee’s self-esteem.

Second, constant high power leads to an increase in self-esteem, which further makes powerful individuals feel special, confident, and privileged. People who are in high power and high-status state for a long time will tend to gradually lose themselves morally. It is important to remind high power individuals regularly that having power does not mean absolute power and privilege, this type of intervention can help reduce the incidence of corruption, violence, and other crimes.

Third, self-defense reinforces the relationship between demotion and lowered self-esteem, which requires the organization or leader to work to make the employee accept the reality of the demotion, reduce his or her sensitivity to the power-decreasing event, and thus reduce the likelihood that he or she will activate self-defense mechanisms. This can be done through a gradual reduction in power, rather than a sudden demotion; or a demotion that is accompanied by a temporary freeze in the employee’s income and benefits.

Fourth, self-regulation is also needed in self-management to reduce one’s adverse reactions to power-decreasing events and to use fewer psychologically immature defense mechanisms so that one does not overreact. Currently, there is a growing group of suicides due to depression, which is predominantly among young people, many of whom have lost hope in life because of setbacks in their lives that have made them devastated. This frustration is the loss of control over the individual’s life and surroundings, i.e., a decrease in power, and therefore, this study may provide theoretical support to alleviate this phenomenon.

### Limitations and directions for future research

The shortcomings of this study and the development of future research are as follows: (1) The manipulation of power decline was a new contextual simulation, and future research is required to validate this intervention more completely. In addition, the context was a company context and the subjects were ordinary college students, which did not ensure that they could empathize with the situation. Future studies could select subjects with a business background, such as MBA students, or set the context of the situation to school. The context could also be modified to fit the specific research context. (2) There are some problems with the representativeness of the subjects. Most of the experimental subjects in this study were selected from student subjects in more economically developed areas. The study confirmed that compared to urban students, rural students are more submissive and more stoic when things go wrong (Liu and Zou, 2015). Therefore, student subjects in economically developed areas may react more intensely to the decline in power, and future research can expand the selection of subjects to take into account factors such as geography and economy. (3) The experimental study with exclusively student subjects has certain shortcomings and cannot fully reflect the impact of power changes in the organizational context, and future studies can add field studies or case studies to further strengthen the validity of the results. (4) The research approach is not sufficiently varied or detailed. Future research can try to design a power decline scale to improve the ecological validity of the study.

## Data availability statement

The raw data supporting the conclusions of this article will be made available by the authors, without undue reservation.

## Ethics statement

The studies involving human participants were reviewed and approved by IRB of College of International Economics and Trade, Ningbo University of Finance and Economics. The patients/participants provided their written informed consent to participate in this study.

## Author contributions

CH: conceived this study, designed questionnaires, collected data, analyzed data, wrote the whole paper and finalized the manuscript for submission.

## Funding

This achievement is partially supported by the “innovative research base for the integration of digital economy and open economy” of Zhejiang soft science research base.

## Conflict of interest

The author declares that the research was conducted in the absence of any commercial or financial relationships that could be construed as a potential conflict of interest.

## Publisher’s note

All claims expressed in this article are solely those of the authors and do not necessarily represent those of their affiliated organizations, or those of the publisher, the editors and the reviewers. Any product that may be evaluated in this article, or claim that may be made by its manufacturer, is not guaranteed or endorsed by the publisher.
